# Green Grouting Material Based on Phosphogypsum–Slag Geopolymer: Feasibility and Performance Evaluation for Trenchless Road Repair

**DOI:** 10.3390/ma18214901

**Published:** 2025-10-26

**Authors:** Xiaoping Ji, Liyuan Dong, Xiaojuan Li, Honglei Lu, Houfu Song, Penghui Wen

**Affiliations:** 1 School of Highway, Chang’an University, Xi’an 710064, China; 2 Technology Development Group Co., Ltd., Shaanxi Transportation Holding Group, Xi’an 710075, China; 3 Department of Automotive Engineering, Guizhou Aerospace Vocational and Technical College, Xinpu Campus, Zunyi 563000, China

**Keywords:** grouting material, phosphogypsum, slag, workability, mechanical strength, durability, toxic substances

## Abstract

Grouting materials are essential for trenchless road repair. However, conventional cement-based grouting materials suffer from considerable shrinkage and low early-age strength. To address these challenges, this study utilizes industrial solid wastes (phosphogypsum and slag) for the synergistic synthesis of a phosphogypsum–slag-based geopolymer (PBG). Using PBG as a binder and fine sand as an aggregate, a sustainable grouting material was developed. The influence of binder-to-sand and water-to-solid ratios on PBG workability was systematically evaluated, identifying the optimal water-to-solid ratio. Based on this, the effects of the binder-to-sand ratio on mechanical strength at various curing ages, durability, and leaching of toxic substances were analyzed. The mechanism of strength development mechanism and immobilization behavior of toxic substances were revealed through SEM. The results indicate that the material exhibits excellent performance when the water-to-solid ratio is 0.28 and the binder-to-sand ratio ranges from 0.70 to 0.75. The material exhibits fluidity of 160–240 mm, initial setting time > 30 min, and final setting time < 400 min, a bleeding rate < 0.4%, and 28-day compressive strength ≥ 9.0 MPa. Both the impermeability and freeze–thaw resistance of PBG grouting material improve with a higher binder-to-sand ratio. Toxic substance leaching complies with Class III groundwater quality standards. Carbon footprint analysis indicates that the material significantly reduces carbon emissions.

## 1. Introduction

With the rapid development of infrastructure in China and the increasing demand for underground engineering, grouting materials have played an irreplaceable role in the reinforcement and rehabilitation of roads, airports, dams, and mining facilities. At present, commonly used grouting materials include cement-based, organic, and chemical grouts. However, conventional cement-based grouting materials have several limitations, including prolonged setting time, high shrinkage, limited durability, and insufficient early-age strength [1,2,3,4]. Therefore, developing novel grouting materials is essential to overcome these limitations. Numerous studies have been conducted to enhance the performance of grouting materials. Sha et al. [5] investigated the durability performance of an effective microfine cementitious grouting material (EMCG) in corrosive environments. They found that the 7-day impermeability pressure of EMCG exceeded 1.0 MPa under sulfate and chloride attack. Compared to conventional cement-based grouting materials, EMCG demonstrated superior long-term stability and corrosion resistance. Li et al. [6] investigated the effects of incorporating nanomaterials into cement-based grouting materials. The results showed that the addition of nanoparticles accelerated the hydration process, while the incorporation of graphene oxide enhanced the resistance of the crack material to failure. The synergistic interaction among different nanomaterials effectively improved the flowability, stability, and mechanical strength of the grouting material. However, most studies still rely on cement as the primary binder, which presents challenges for reducing environmental impact and improving the efficiency of solid waste resource utilization. Although organic grouting materials exhibit favorable expansion properties, impermeability, and mechanical strength [7,8,9], their high cost and poor environmental compatibility [10] have limited their widespread application in large-scale engineering projects.

The accumulation of industrial solid wastes in China has become increasingly severe, with the disposal of phosphogypsum and slag presenting particularly significant challenges. Phosphogypsum is a by-product generated during the wet-process production of phosphoric acid, with 4 to 6 tons of waste phosphogypsum produced for every ton of phosphoric acid synthesized [11,12]. It primarily consists of calcium sulfate dihydrate (CaSO_4_ 2H_2_O) and contains various pollutants, including soluble phosphates, fluorides, and heavy metals. Due to the potential mobility of these hazardous substances, phosphogypsum is classified as an industrial solid waste with significant environmental risks [13,14]. The global annual production of phosphogypsum exceeds 280 million tons. In China, the accumulated stockpile reached 870 million tons by 2024, with a yearly increase of approximately 80 million tons and a comprehensive utilization rate of less than 40%. The open-air stockpiling of phosphogypsum not only occupies substantial land resources but also poses environmental risks due to the leaching of contaminants [15]. Current utilization pathways primarily include applications as cement retarders [16], gypsum-based building materials [17], mine backfill materials [18], and soil conditioners [19]. However, these approaches are limited by low utilization rates and minimal added value. With the goals of carbon peaking and carbon neutrality, achieving large-scale and high-value utilization of phosphogypsum has become a critical issue that requires urgent attention. Slag, a by-product generated during blast furnace ironmaking in the metallurgical industry, is primarily composed of CaO, SiO_2_, and Al_2_O_3_. Based on the cooling method used, slag can be classified into water-quenched granulated blast slag and air-cooled slag [20]. Among these, water-quenched slag exhibits higher reactivity due to its higher glassy phase content. As of 2024, China’s annual slag production capacity has reached 345 million tons, with the majority used as a supplementary cementitious material in cement and concrete. The incorporation of slag can significantly reduce CO_2_ emissions in the cement industry while enhancing the long-term strength and durability of concrete [21,22]. Slag exhibits a high pozzolanic activity and can react with Ca(OH)_2_ under alkaline conditions to form calcium silicate hydrate (C-S-H) gel [23]. However, its slow early-age strength development, need for fine grinding, and high activation requirements still limit its broader application.

Geopolymers were first discovered by French professor Davidovits [24]. Due to their rapid strength development, low cost, wide availability of raw materials, and environmentally friendly characteristics, geopolymers have emerged as ideal materials for road repair. With the growing awareness of sustainable development and the widespread adoption of low-carbon and eco-friendly concepts, the application of geopolymers has expanded. Feng et al. [25] developed geopolymer concrete (PGC) by partially replacing blast furnace slag with phosphogypsum. The results indicated that incorporating 10% phosphogypsum significantly promoted the formation of C-(A)-S-H gel and enhanced the material’s microstructure, resulting in an approximately 17% increase in axial compressive strength and a notable reduction in the chloride ion diffusion coefficient. These findings confirmed the positive effect of phosphogypsum in promoting the geopolymerization process. Oubaha et al. [26] synthesized acid-activated geopolymers using a combination of phosphogypsum and phosphate clay. Phosphoric acid was used as the activator, and the optimal formulation, determined via response surface methodology, consisted of 28.83% phosphogypsum, a phosphoric acid concentration of 14.39 mol/L, and a curing temperature of 72.45 °C. The resulting material achieved a 28-day compressive strength of 21.46 MPa, with no detectable leaching of hazardous substances. Adebayo et al. [27] evaluated the strength and hardness of geopolymer concrete made from fly ash and slag. The study involved partially replacing natural sand with varying percentages of crumb rubber (CR) obtained from discarded tires, as well as incorporating polyethylene fibers (PEF) at percentages ranging from 0.25% to 0.75%, to systematically examine their effects on material performance. The experimental results demonstrated a significant synergistic enhancement between CR and PEF. Compared to single-additive systems, the composite mixture containing 5% CR and 0.75% PEF performed better overall in terms of compressive strength, ductility, and elastic modulus. Similarly, in the resource utilization of industrial solid wastes, the study by Cherrat et al. [28] found that FTP dust recovered from electric arc furnaces, after coal treatment, can efficiently recover refined zinc, further expanding the feasibility of high-value circular applications for industrial waste.

Currently, the utilization of solid wastes for the preparation of geopolymer grouting materials is in its early stages. In particular, systematic investigations into slurry workability, strength development mechanisms, and environmental safety remain relatively limited. The synergistic use of phosphogypsum and slag to prepare geopolymer-based grouting materials for road repair has the potential to overcome the inherent limitations of typical cement-based materials. Furthermore, it facilitates the recycling of industrial waste resources, providing both significant environmental benefits and economic advantages.

In this study, the alkali activation technique was employed to activate PBG through the synergistic utilization of phosphogypsum and slag. A PBG grouting material was developed for application in various trenchless road repair scenarios, with PBG as the binder and fine sand as the aggregate. This study examined the effects of binder-to-sand ratio and water-to-solid ratio on the workability of the grouting material. Building on the optimal water-to-solid ratio, the impact of the binder-to-sand ratio on the material’s mechanical strength, durability, and leaching of toxic substances was further examined. The strength development mechanism and chemical immobilization behavior of toxic constituents in the PBG grouting material were analyzed through Scanning Electron Microscopy (SEM). Additionally, a carbon footprint analysis was performed, and the optimal mix design for the PBG grouting material was recommended for practical application. The research outcomes contribute to the advancement of the resource-efficient and high-value utilization of phosphogypsum and slag.

## 2. Materials and Methods

### 2.1. Raw Materials

#### 2.1.1. Phosphogypsum–Slag-Based Geopolymer

The PBG used in this study is a green binder synthesized from phosphogypsum (Sichuan, China) and slag (Sichuan, China) under alkali activation (Sichuan, China). Based on preliminary systematic experimental studies across a range of parameters, including the phosphogypsum/slag ratio (4:6 to 7:3), alkali activator dosage (10% to 16%), alkali activator modulus (1.0 to 1.6), and water-to-solid ratio (0.48 to 0.54), the optimal mix design was ultimately determined. The optimal mix consists of a phosphogypsum-to-slag mass ratio of 1:1, an alkali activator dosage of 16%, an alkali activator modulus of 1.2, and a water-to-solid ratio of 0.52 (Table 1). Under these conditions, the prepared PBG material demonstrates excellent mechanical properties. Quantitative analyses of heavy metal elements and water-soluble anions in PBG were performed by Inductively Coupled Plasma Optical Emission Spectrometry (ICP-OES) and Ion Chromatography (IC), respectively. The results demonstrate that the leaching concentrations of toxic substances comply with the limits specified in the relevant secondary standards [29]. The key technical properties of the PBG are summarized in Table 2.

#### 2.1.2. Fine Sand

**Figure 1:**
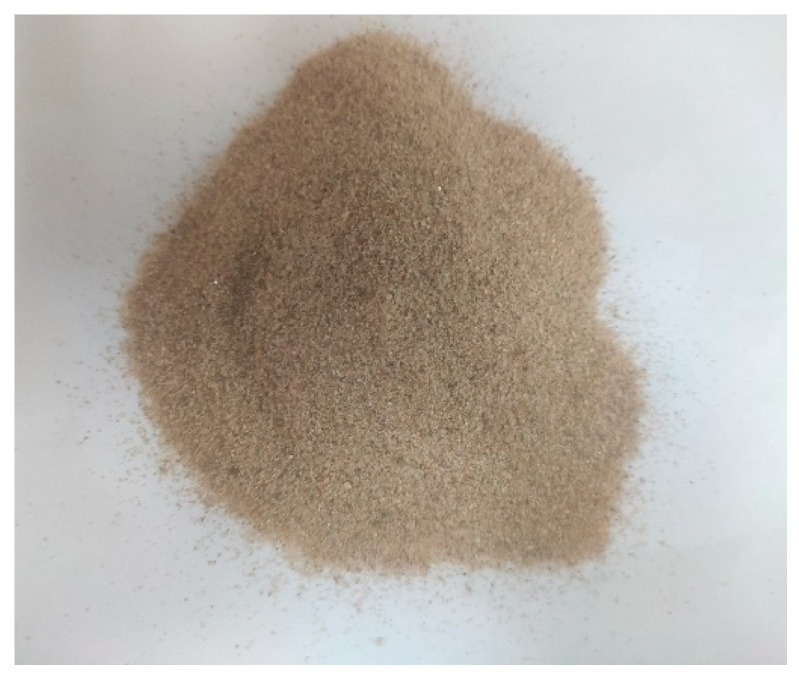
Fine sand was used in the experiment.

**Figure 2:**
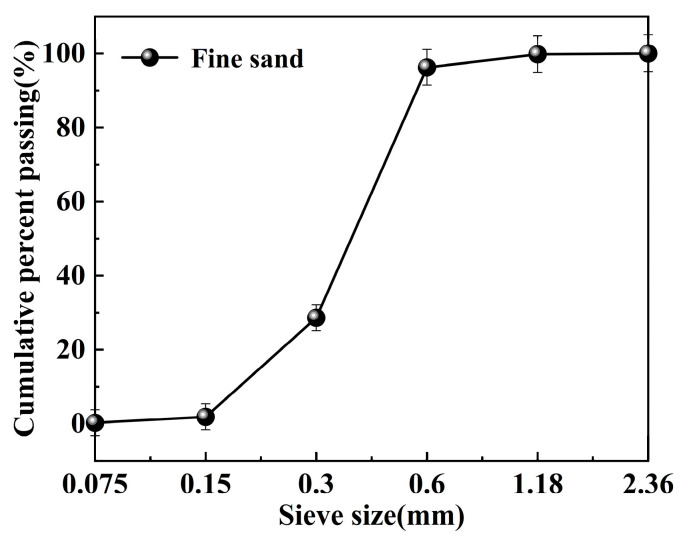
Particle size distribution of fine sand.

The physical properties of fine sand (Sichuan, China) are key factors in determining its strength [30]. The morphology of the fine sand used in the experiment is shown in Figure 1. Its bulk density is 1.423 g/cm^3^, apparent density is 2.491 g/cm^3^, and mud content is 2.0%. The particle size distribution ranges from 0.15 to 1.18 mm, as illustrated in Figure 2.

### 2.2. Test Methods

#### 2.2.1. Workability

##### Flowability

**Figure 3:**
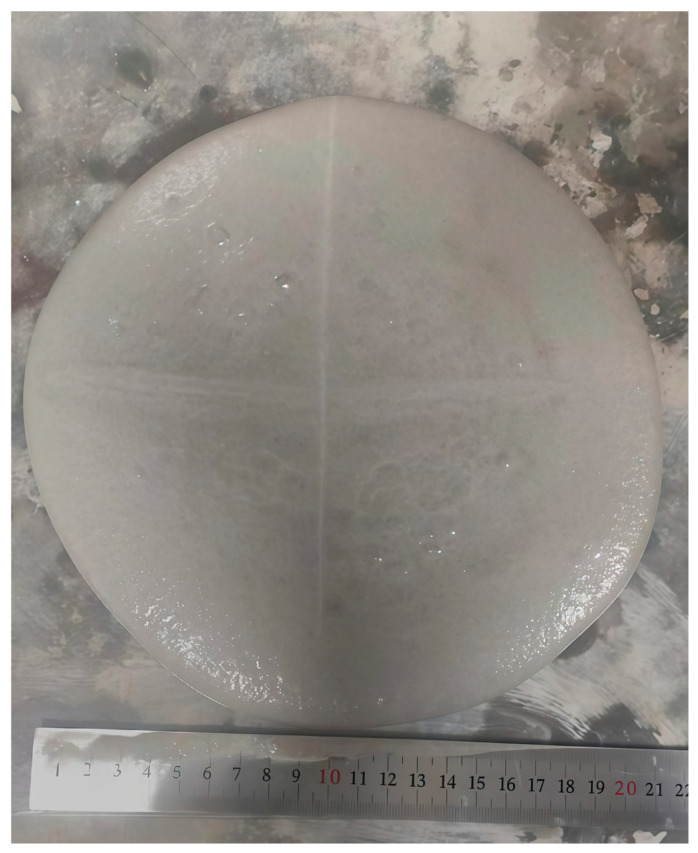
Fluidity test.

The fluidity of the PBG grouting material was evaluated using a smooth, hollow glass cylinder with both height and diameter measuring 80 mm. The specific steps were as follows: first, the clean and dry hollow cylinder was placed vertically at the geometric center of a square glass base plate. Place the pre-weighed phosphogypsum, slag, and fine sand into a dry cement paste mixing bowl, and start the mixing program. Mix at a speed of 62.5 r/min for 2 min to ensure the base materials are evenly blended. Then, add the pre-prepared alkaline activator and mix again at 62.5 r/min for 4 min to ensure the slurry is thoroughly mixed. Next, the well-mixed PBG slurry was steadily poured into the cylinder until the liquid surface was level with the top edge of the mold. The cylinder was then quickly lifted vertically, allowing the slurry to collapse and spread on the base plate. The spread pattern was observed, and the flow diameters in both the horizontal and vertical directions were measured. Finally, the arithmetic mean of the vertical and horizontal diameters was taken as the fluidity result, as shown in Figure 3.

##### Setting Time

**Figure 4:**
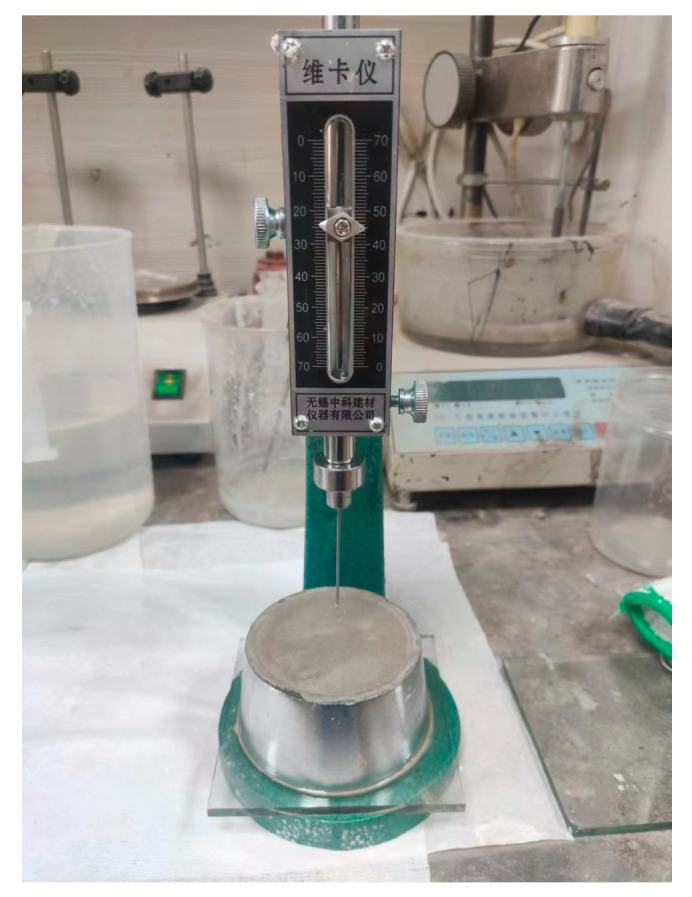
Setting time test.

The setting time of PBG grouting material is a critical parameter influencing its construction workability and must be optimized according to specific engineering requirements. If the setting time is too short, the material may lose its flowability during long-distance transport and on-site application. Conversely, an excessively long setting time can significantly extend the curing period and adversely affect construction progress. In accordance with the specification requirements [31], the initial setting time shall be ≥30 min, and the final setting time shall be ≤400 min. Experimental measurements and rationality evaluation were conducted on the setting characteristics of the PBG grouting material to evaluate whether its workability meets the standards required for practical engineering applications. This study employed the Vicat apparatus (Sichuan, China) to determine the initial setting time and final setting time of the PBG grouting material, as illustrated in Figure 4.

##### Bleeding Rate

**Figure 5:**
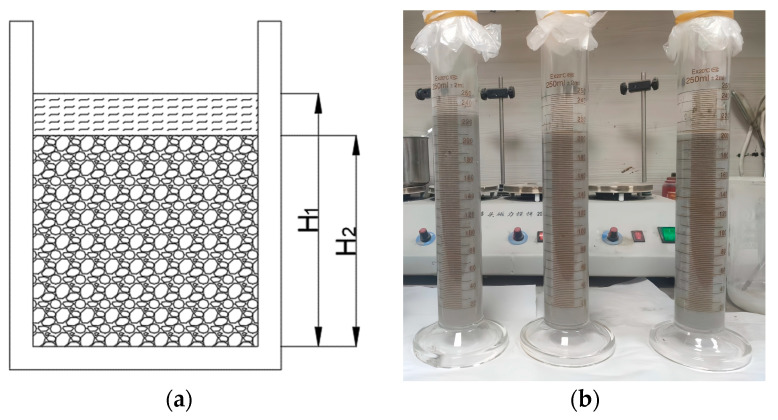
Bleeding rate test: (**a**) The schematic diagram of the bleeding rate test; (**b**) specimen and container.

The bleeding rate of the PBG grouting material is a critical indicator for evaluating its workability and stability, as it directly influences the material’s segregation resistance and its eventual mechanical strength. To quantitatively characterize its bleeding behavior, the testing method adopted in this study is illustrated in Figure 5. The freshly mixed PBG slurry was poured into a graduated glass cylinder with a known volume, and the initial height of the slurry was recorded as H_1_. The top of the cylinder was then sealed with a polyethylene film to prevent moisture evaporation and external contamination. After standing for 2 h, the actual height of the slurry, excluding the bleeding layer, was measured as H_2_. The bleeding rate was subsequently calculated according to Equation (1). In accordance with the standard requirements [31], the bleeding rate of the PBG grouting material was assessed to verify its compliance with the technical requirement of not exceeding 0.40%. To ensure the accuracy and reliability of the experimental results, three parallel specimens were prepared for each sample. All parallel specimens were fabricated and tested simultaneously under strictly identical conditions.

(1) $$\mathrm{Bleeding}\;\mathrm{rate}=\frac{H_{1}-H_{2}}{H_{1}}\times 100\%$$

where H_1_ is the original height of the material in its initial state (mm); H_2_ is the actual height of the slurry after excluding the bleeding layer (mm).

#### 2.2.2. Mechanical Strength

Determining the mechanical strength of PBG grouting material is a critical step in ensuring its engineering applicability. The strength parameters directly reflect the load-bearing capacity and structural stability of the material under applied loads, providing essential data for mix design optimization, construction quality control, and long-term durability prediction, thereby ensuring the safety and cost-effectiveness of engineering applications. As required by the specification [31], the 28 days compressive strength of the PBG grouting material was evaluated to verify its compliance with the specification requirement of no less than 4.2 MPa.

**Figure 6:**
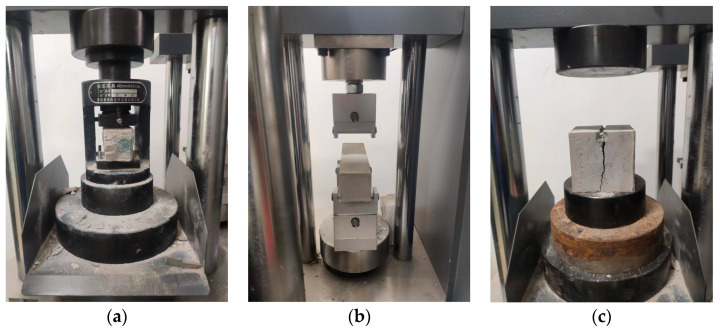
Mechanical strength test of PBG grouting material: (**a**) Compressive strength; (**b**) flexural strength (**c**) Splitting tensile strength.

The test methods were carried out in accordance with the current specifications for highway engineering [32]. The PBG grouting slurry was cast into standard triple-gang molds to prepare prismatic specimens with dimensions of 40 mm × 40 mm × 160 mm and cubic specimens measuring 70.7 mm × 70.7 mm × 70.7 mm. The specimens were cured in a standard curing box at a temperature of 20 ± 2 °C and a relative humidity of 95% or higher. The prismatic specimens were used for flexural strength testing, and the resulting halves from the fractured specimens were subsequently utilized for compressive strength testing. The cubic specimens were employed to evaluate the splitting tensile strength. The mechanical strength tests were conducted in strict accordance with the principle of parallel repetition. For each sample, three parallel specimens were prepared, and the arithmetic mean was calculated after analyzing the data dispersion, thereby ensuring the scientific validity and reliability of the test results. All mechanical strength tests were conducted using a universal testing machine with a constant displacement rate of 0.5 mm/min. The testing setup is illustrated in Figure 6.

#### 2.2.3. Durability

##### Impermeability

The impermeability of geopolymer grouting materials is a critical indicator of their resistance to pressurized water infiltration. It plays a vital role in road rehabilitation and serves as a key parameter for evaluating the material’s durability. During the hydration process of geopolymer grouting materials, interconnected microcracks and capillary pores may develop within the internal structure. In subsurface environments, pressurized water can infiltrate the material through these microcracks and pores, further promoting crack propagation and potentially leading to structural failure. This deterioration significantly compromises the material’s durability. Therefore, an in-depth investigation into the impermeability of geopolymer grouting materials is crucial.

**Figure 7:**
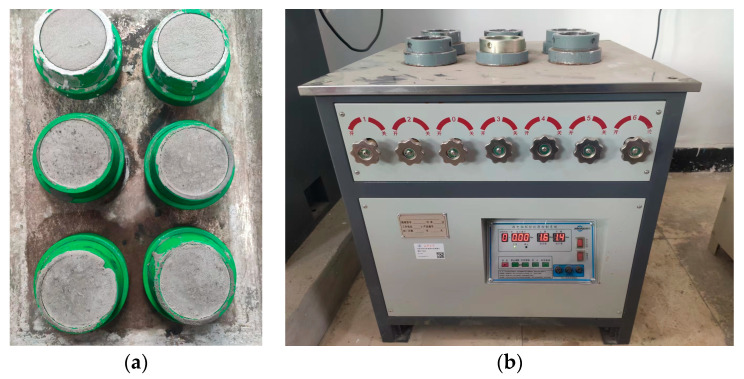
Impermeability test of PBG grouting material: (**a**) Specimens for impermeability testing; (**b**) mortar permeability apparatus.

The freshly prepared PBG grouting slurry was cast into frustum-shaped molds with diameters of 7 cm and 8 cm and a height of 3 cm. The specimens were then placed in a standard curing environment and maintained until the designated curing age was reached. After curing, the specimens were coated with silicone sealant along their sidewalls and installed into the mortar permeability testing apparatus. The edges of the specimens were then resealed with silicone to ensure watertightness and prevent leakage during the testing process. Once the silicone sealant had fully cured, the permeability test was conducted on the PBG grouting material specimens, as illustrated in Figure 7. During the test, the initial water pressure of the mortar permeability apparatus was set to 0.2 MPa. After 2 h, the apparatus automatically increased the pressure to 0.3 MPa, followed by incremental increases of 0.1 MPa every hour. The water pressure at which seepage was first observed at the top of the specimen was recorded, and the impermeability pressure of the specimen was calculated using Equation (2).

(2) P=H−0.1

where *P* is impermeability pressure of the specimen (MPa); *H* is water pressure at the time seepage occurred during the test (MPa).

##### Freeze–Thaw Resistant

**Figure 8:**
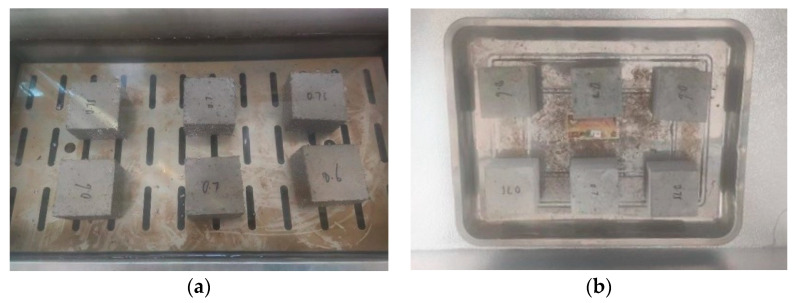
Freeze–thaw test of PBG grouting material: (**a**) Specimen water soaking (**b**) Specimen freezing.

When used for road rehabilitation, grouting materials may be subjected to alternating cycles of groundwater exposure and temperature fluctuations, which can potentially lead to freeze–thaw damage. In this study, cubic specimens with dimensions of 70.7 mm × 70.7 mm × 70.7 mm were prepared by casting and molding, and subsequently cured for 28 days under standard curing conditions. After curing, the specimens were immersed in water at 20 °C for 48 h. Upon completion of soaking, they were transferred to a low-temperature chamber pre-cooled to −15 °C for freezing over a period of 4 h. The frozen specimens were then removed and placed in water at approximately 20 °C for 4 h to undergo thawing. This process constituted one complete freeze–thaw cycle, as illustrated in Figure 8. The compressive strength of the specimens was recorded after different numbers of freeze–thaw cycles. Based on these results, the BDR (Bonding Durability Ratio) of the PBG grouting material was calculated using Equation (3).

(3) $$BRD=\frac{R_{DC}}{R_{C}}\times 100$$

where *BDR* is the residual strength ratio of the specimen (%); *R_DC_* is the compressive strength of the specimen after *n* freeze–thaw cycles (MPa); *R_C_* is the standard compressive strength of the specimen (MPa).

#### 2.2.4. Leaching of Toxic Substances

Phosphogypsum contains harmful components, including soluble phosphorus, fluorine, and trace heavy metals, which pose a potential risk of groundwater contamination when PBG grouting material is applied in road engineering. The leaching behavior of toxic substances was evaluated by simulating natural environmental conditions to determine the concentration of released pollutants and assess the material’s environmental safety.

**Figure 9:**
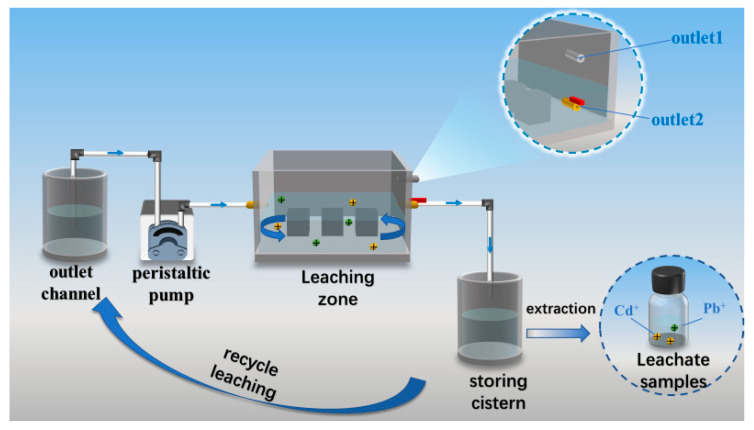
Dynamic cycle leaching device for toxic substances.

A self-developed dynamic cyclic leaching apparatus, designed by the research team, was employed for the testing, as illustrated in Figure 9. The leaching of toxic substances from the PBG grouting material was simulated under groundwater conditions [33]. The test system was composed of key components, including a leaching chamber, a water storage tank, an effluent collection tank, and a peristaltic pump. During the experiment, a strict solid-to-liquid ratio of 1:20 was maintained, with distilled water used as the leaching medium. The specific testing procedure was as follows: first, the specimens were placed in the leaching chamber with a minimum spacing of at least 10 mm between them. A measured volume of distilled water was then introduced into the effluent tank and rapidly pumped into the leaching chamber via a peristaltic pump until the liquid level reached the height of outlet port two. Next, the pump was adjusted to a low-speed replenishment mode, while outlet port two was simultaneously opened to allow the leachate to flow by gravity into the storage tank. Once the effluent tank was emptied, the leachate collected in the storage tank was returned entirely to the effluent tank, completing one leaching cycle. The above leaching cycle was repeated multiple times to ensure the reliability and reproducibility of the test data.

Finally, the concentrations of heavy metal elements (As, Cr, Pb) in the leachate were quantitatively analyzed using Inductively Coupled Plasma Optical Emission Spectroscopy (ICP-OES), while the leaching amounts of water-soluble anions (F^−^, PO_4_^3−^) were determined using Ion Chromatography (IC). All test results were evaluated with reference to the Class III groundwater standard limits [29]. Specifically, the leachate concentrations must not exceed 10 μg/L for arsenic (As) and lead (Pb), 50 μg/L for chromium (Cr), and 1 mg/L for fluoride (F^−^).

### 2.3. Experimental Design Matrix

Based on a multi-factor experimental design, this study comprehensively evaluates the workability, mechanical strength, durability, and environmental characteristics of the PBG grouting material. The specific variables and levels matrix is shown in Table 3.

**Figure 10:**
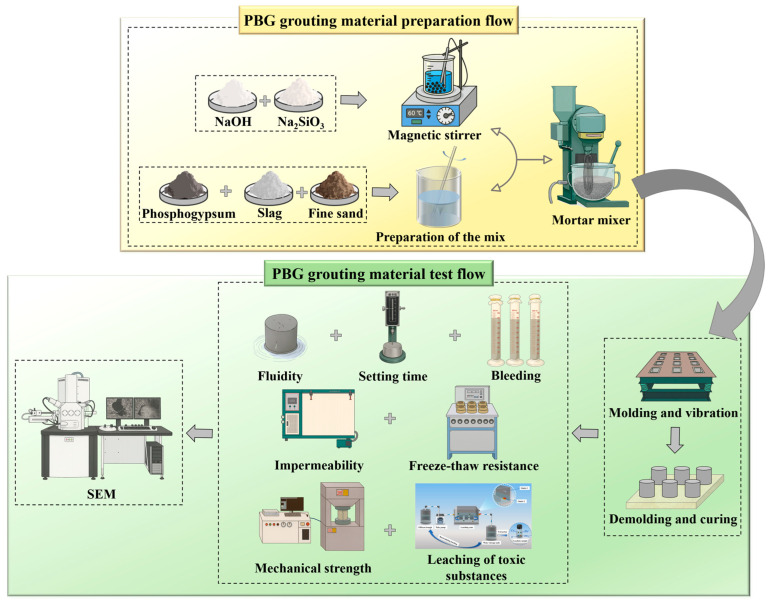
Schematic flowchart of PBG grouting material.

The preparation and evaluation process of PBG grouting material is shown in Figure 10.

## 3. Results and Discussions

### 3.1. Workability of PBG Grouting Material

#### 3.1.1. Flowability

**Figure 11:**
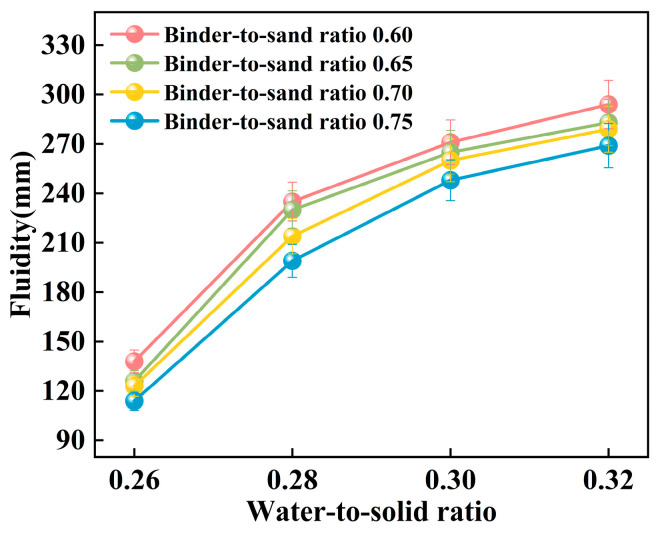
Relationship between the fluidity and the water-to-solid ratio.

**Figure 12:**
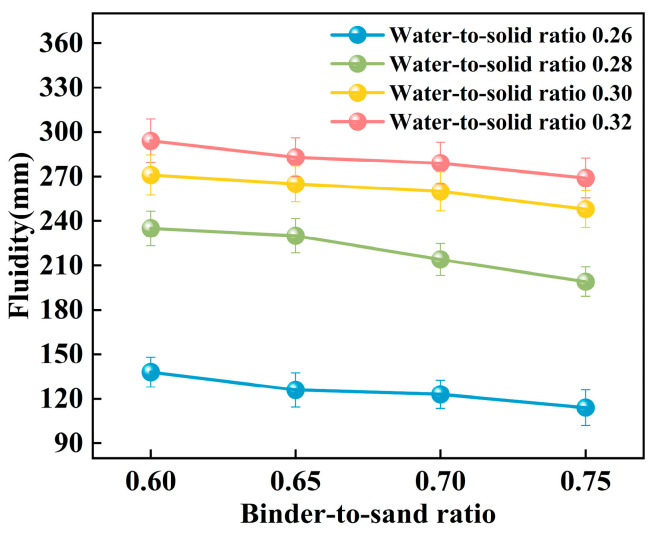
Relationship between the fluidity and the binder-to-sand ratio.

The experiment systematically investigated the influence of binder-to-sand ratio and water-to-solid ratio on the fluidity of PBG grouting material. The binder-to-sand ratio (defined as the mass ratio of PBG binder to fine sand) was set at four levels: 0.60, 0.65, 0.70, and 0.75, while the water-to-solid ratio was varied across four levels: 0.26, 0.28, 0.30, and 0.32. The experimental results are presented in Figure 11 and Figure 12.

The experimental results presented in Figure 11 indicate that the fluidity of PBG grouting material exhibits a significant positive correlation with the water-to-solid ratio. Specifically, under binder-to-sand ratios of 0.60, 0.65, 0.70, and 0.75, the fluidity of the PBG grouting material increases markedly as the water-to-solid ratio rises from 0.26 to 0.32. Correspondingly, the measured fluidity values increase from 138 mm, 126 mm, 123 mm, and 114 mm to 294 mm, 284 mm, 279 mm, and 269 mm, respectively. It is noteworthy that when the water-to-solid ratio is maintained at 0.28, the fluidity under all binder-to-sand ratio conditions remains within the range of 160 mm to 240 mm, meeting the technical requirements specified in relevant standards. This observation underscores the significant impact of the water-to-solid ratio on the workability of PBG grouting material.

Figure 12 illustrates the influence of the binder-to-sand ratio on the fluidity of PBG grouting material. Experimental results indicate that, under a constant water-to-solid ratio, fluidity exhibits a negative correlation with the binder-to-sand ratio. This phenomenon can be attributed to the following mechanism: An increase in the binder-to-sand ratio results in a higher proportion of PBG binder within the system, which accelerates the hydration reaction process. As a result, it facilitates the generation of abundant gel products and the formation of a dense three-dimensional network structure, thereby strengthening interparticle bonding among raw materials and ultimately reducing the slurry’s fluidity [34,35]. Furthermore, as the binder-to-sand ratio increases, the content of fundamental binder components such as phosphogypsum and slag powder in the slurry also rises. Compared to aggregate sand particles, these binder materials possess a higher specific surface area, greater water absorption capacity, and a rougher surface texture. These physical characteristics collectively contribute to a further decline in the slurry’s fluidity.

#### 3.1.2. Setting Time

**Figure 13:**
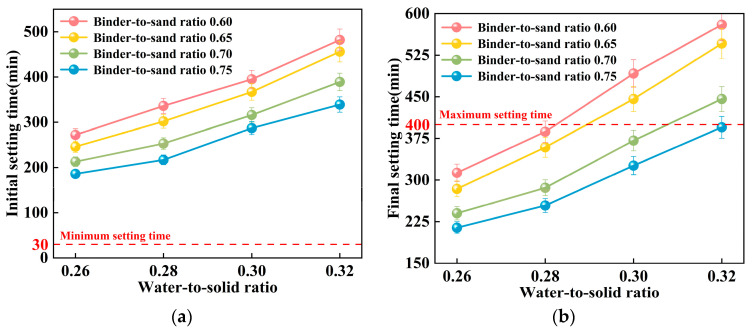
Relationship between setting time and water-to-solid ratio of PBG grouting material: (**a**) Relationship between initial setting time and water-to-solid ratio (**b**) Relationship between final setting time and water-to-solid ratio.

**Figure 14:**
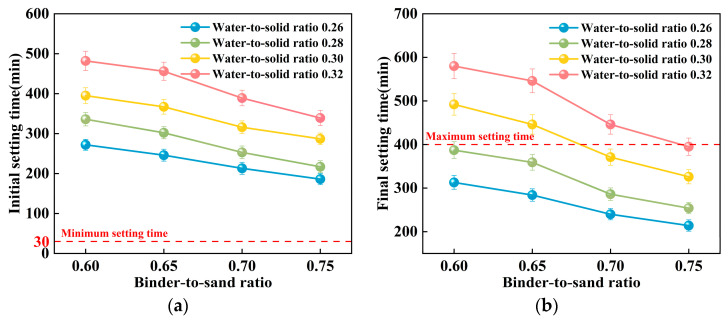
Relationship between the setting time of PBG grouting material and the binder-to-sand ratio: (**a**) Relationship between initial setting time and binder-to-sand ratio (**b**) Relationship between final setting time and binder-to-sand ratio.

This study examines the impact of the binder-to-sand ratio and water-to-solid ratio on the setting time of PBG grouting material. The binder-to-sand ratios were set at 0.60, 0.65, 0.70, and 0.75, and the water-to-solid ratios were set as 0.26, 0.28, 0.30, and 0.32. The experimental results are presented in Figure 13 and Figure 14.

As shown in Figure 13, the water-to-solid ratio has a significant influence on the setting time of PBG grouting material. Experimental results indicate that both the initial and final setting times increase progressively with an increase in the water-to-solid ratio. When the water-to-solid ratio is maintained within the range of 0.26 to 0.28, the initial setting time reaches a minimum of 186 min, while the final setting time does not exceed 336 min. These values comply with the technical requirements specified in [31], which require an initial setting time of at least 30 min and a final setting time of no more than 400 min. When the water-to-solid ratio is increased to 0.30, specimens with binder-to-sand ratios of 0.70 and 0.75 also meet the specified technical requirements. Mechanism analysis reveals that, at lower water-to-solid ratios, the reaction system exhibits relatively high concentrations of dissolved silicon (Si) and aluminum (Al) species. These elevated ionic concentrations effectively enhance both the geopolymerization and hydration processes. Consequently, the setting of the slurry is substantially accelerated. In contrast, higher water-to-solid ratios lead to a dilution of alkali activator concentrations within the reaction system. This dilution inhibits the formation of a well-developed three-dimensional polymeric network structure. As a consequence, the setting time of the slurry is significantly prolonged. This phenomenon clearly demonstrates the regulatory effect of the water-to-solid ratio on the reaction kinetics of the geopolymer system.

Figure 14 illustrates the influence of the binder-to-sand ratio on the setting time of PBG grouting material. Experimental results indicate that both the initial and final setting times of the PBG grouting material decrease significantly with the increase in binder-to-sand ratio. Specifically, at a water-to-solid ratio of 0.28, increasing the binder-to-sand ratio from 0.60 to 0.75 results in a pronounced decrease in the initial setting time. The setting time is reduced from 336 min to 217 min, indicating a significant enhancement in the early reaction kinetics. This phenomenon can be interpreted from two mechanistic perspectives. First, increasing the binder-to-sand ratio elevates the proportion of silico-aluminous precursors (slag) within the reactive matrix. This compositional change facilitates the formation of a greater number of multiphase nucleation sites during the initial depolymerization stage of geopolymerization. As a result, the setting rate of the slurry is effectively accelerated. Second, the elevated concentrations of dissolved [SiO_4_]^4−^ and [AlO_4_]^5−^ species in the reaction system promote their polymerization with free Ca^2+^ ions. This interaction facilitates the development of a three-dimensional gel network. The network is primarily composed of interconnected [SiO_4_] (Silicate tetrahedra) and [AlO_4_] (Aluminate tetrahedra), which contribute to the structural integrity of the hardened matrix [36]. This structural development significantly shortens the setting time of the slurry.

#### 3.1.3. Bleeding Rate

**Figure 15:**
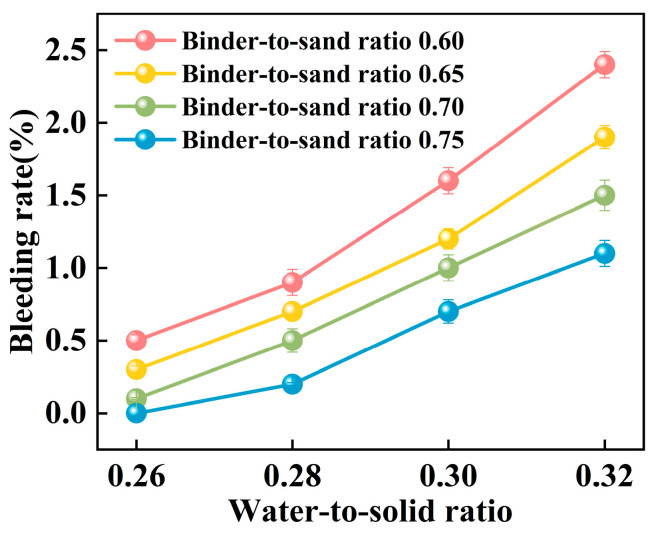
Relationship between bleeding rate and water-to-sand ratio.

**Figure 16:**
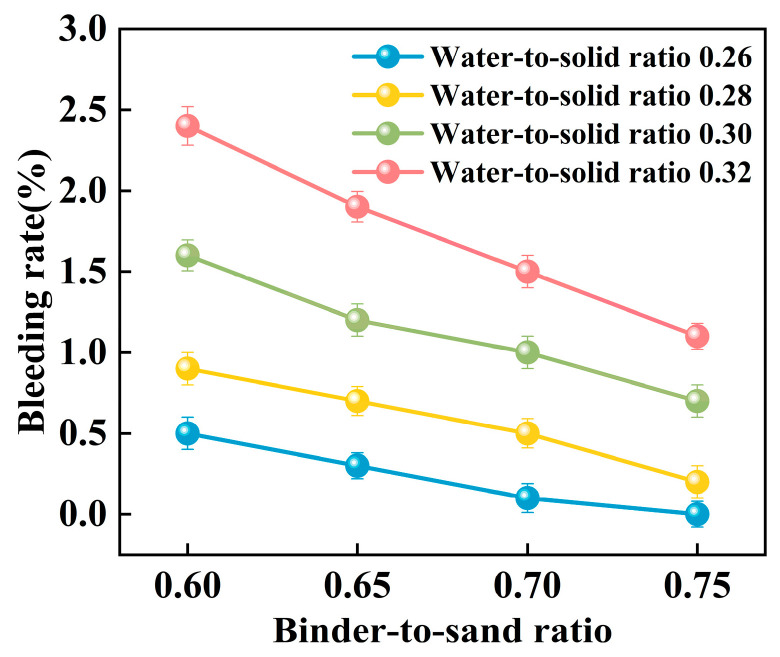
Relationship between bleeding rate and binder-to-sand ratio.

Experimental investigations were conducted to elucidate the influence of the binder-to-sand ratio and water-to-solid ratio on the bleeding behavior of PBG grouting material. The binder-to-sand ratios were set at 0.60, 0.65, 0.70, and 0.75, while the water-to-solid ratios were selected as 0.26, 0.28, 0.30, and 0.32. The results are presented in Figure 15 and Figure 16.

The experimental results indicate a significant positive correlation between the water-to-solid ratio and the bleeding rate of PBG grouting material (Figure 15). At a water-to-solid ratio of 0.28 and binder-to-sand ratios of 0.70 and 0.75, the bleeding rate of the PBG grouting material ranged from 0% (indicating no observable bleeding) to 0.40%. These values fully comply with the technical requirements for engineering applications, which specify a maximum allowable bleeding rate of less than 0.40%. This result confirms the excellent stability of the material under the given mix proportions. It is noteworthy that, under a constant binder-to-sand ratio, the bleeding rate increases monotonically with the increase in the water-to-solid ratio. When the water-to-solid ratio reaches 0.28, a pronounced increase in the bleeding rate is observed. The phenomenon indicates that the free water content in the system has surpassed the critical saturation threshold. As a result, excess water tends to separate from the slurry, thereby reducing its stability. The surpassing reduces the capacity of solid particles within the slurry to retain water, thereby triggering a nonlinear increase in bleeding behavior. This phenomenon can be explained from a microscopic perspective: An increase in the water-to-solid ratio directly raises the proportion of free water within the slurry system. The resulting excess water diminishes the interparticle interactions among solid constituents. As a consequence, the material exhibits a significant change in bleeding behavior, indicating compromised stability.

As shown in Figure 16, the binder-to-sand ratio exhibits a pronounced and systematic influence on the bleeding rate of PBG grouting material. Experimental results indicate a negative correlation between the bleeding rate and the binder-to-sand ratio. As the binder-to-sand ratio increases, the bleeding rate declines significantly. This trend suggests that a higher binder-to-sand ratio contributes to improved slurry stability by reducing the tendency for water separation. Specifically, under a constant water-to-solid ratio, increasing the binder-to-sand ratio from 0.60 to 0.75 resulted in a reduction in the bleeding rate from 0.5%, 0.9%, 1.6%, and 2.4% to 0%, 0.2%, 0.7%, and 1.1%. This phenomenon can be attributed to the following mechanisms: First, an increased binder-to-sand ratio accelerates the geopolymerization process, promoting the rapid consumption of free water within the slurry, thereby suppressing bleeding. Secondly, due to their finer particle size and larger specific surface area, fundamental materials such as phosphogypsum and slag exhibit stronger water absorption capacity. Compared with sand particles, they are more effective in retaining free water within the slurry, thereby significantly reducing the bleeding rate.

In summary, the water-to-solid ratio is a crucial factor that influences the workability of PBG grouting material. Research indicates that a higher water-to-solid ratio leads to an increase in the thickness of the water film surrounding particle surfaces within the slurry. The presence of a thicker water film reduces interparticle cohesion and internal friction. The overall flowability of the slurry is enhanced. However, excessive free water may lead to an elevated risk of segregation and a prolonged setting time. Conversely, a lower water-to-solid ratio can significantly improve the slurry’s water retention and stability, but the resulting increase in viscosity tends to reduce its flowability. Based on the analysis of experimental data, the PBG grouting material demonstrates favorable performance at a water-to-solid ratio of 0.28. Specifically, the flowability falls within the range of 160–240 mm, the initial setting time exceeds 30 min, and the final setting time remains below 400 min. Additionally, the bleeding rate is substantially lower than 0.40%, indicating excellent slurry stability at this mix ratio. Therefore, this study identifies 0.28 as the optimal water-to-solid ratio, as it ensures excellent workability.

### 3.2. Mechanical Strength of PBG Grouting Material

In this study, the effects of binder-to-sand ratio (0.60, 0.65, 0.70, and 0.75) and curing age on the mechanical strength of PBG grouting material were systematically investigated under a fixed water-to-solid ratio of 0.28. Specifically, the compressive strength, flexural strength, and splitting tensile strength were measured at curing ages of 3 days, 7 days, 14 days, and 28 days to evaluate the strength development characteristics of the material.

#### 3.2.1. Compressive Strength

**Figure 17:**
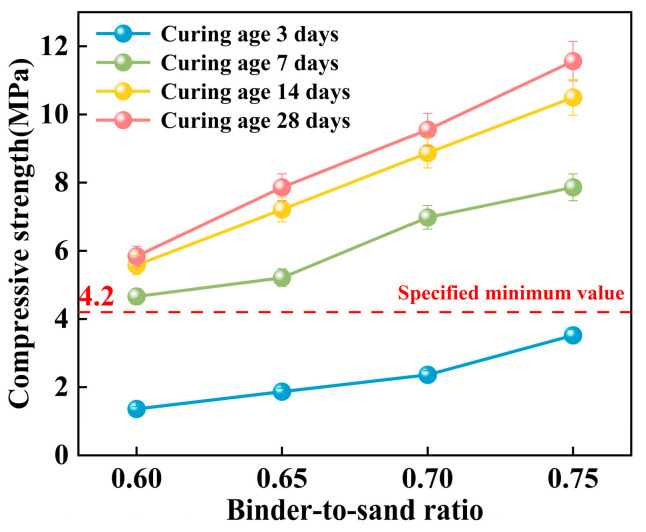
Effect of binder-to-sand ratio on compressive strength.

As shown in Figure 17, the compressive strength of the PBG grouting material exhibits a significant positive correlation with the binder-to-sand ratio. Experimental results indicate a substantial enhancement in the 28 days compressive strength of the PBG grouting material with increasing binder-to-sand ratio. Specifically, the compressive strength increases from 5.84 MPa at a binder-to-sand ratio of 0.60 to 11.56 MPa at a ratio of 0.75. This corresponds to a 97.9% improvement, highlighting the significant positive effect of higher binder content on mechanical performance. Notably, the 28 days compressive strength of the PBG grouting material exceeded the minimum technical requirement [31] of 4.2 MPa at all tested binder-to-sand ratios. This increasing strength trend can be attributed to the higher PBG content resulting from the elevated binder-to-sand ratio, which enhances the bonding between hydration gel products and aggregate interfaces. Accordingly, the internal three-dimensional network structure is optimized, resulting in improved material compactness and contributing to an enhancement of the macroscopic mechanical strength.

**Figure 18:**
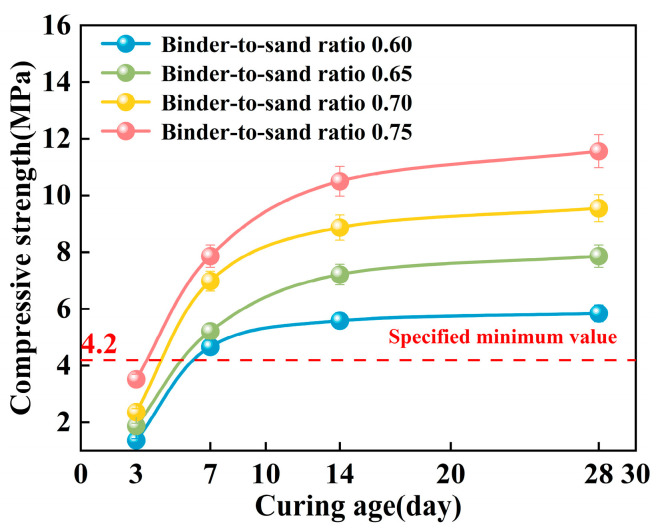
Effect of curing age on compressive strength.

As shown in Figure 18, the compressive strength of the PBG grouting material exhibits a distinct staged growth pattern with increasing curing age. Under a constant binder-to-sand ratio, the relationship between compressive strength and curing time follows a logarithmic function. Specifically, strength increases most significantly within the 0–7 days period, the growth rate gradually declines between 7 and 14 days, and the strength development tends to plateau after 28 days. This phenomenon can be attributed to the kinetic characteristics of the geopolymer hydration reaction. In the early stages of the reaction, high chemical activity facilitates the rapid generation of a substantial quantity of hydration gel products. The formation of these gels promotes the densification of the three-dimensional network structure within the matrix. Consequently, a notable increase in mechanical strength is observed during the initial stage of the curing process. As the curing age extends, the available reactive components within the system are progressively consumed. This gradual depletion limits the continued formation of hydration products. The rate of strength development decreases during the later stages of the curing process. This evolutionary trend is consistent with the typical hydration behavior of alkali-activated cementitious materials.

#### 3.2.2. Flexural Strength

**Figure 19:**
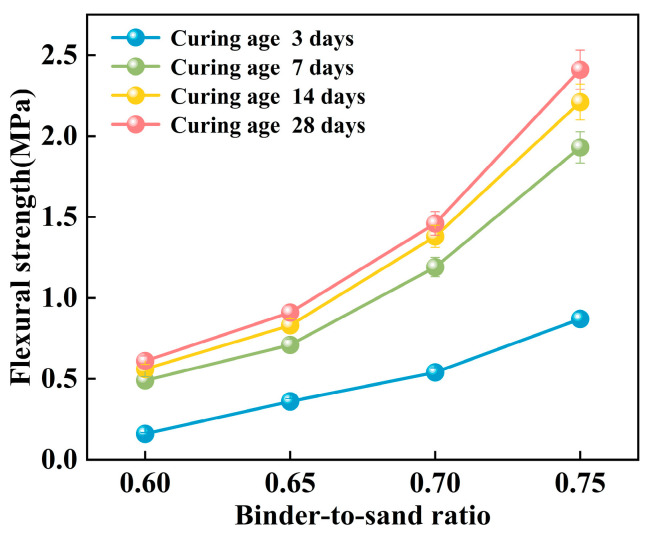
Effect of binder-to-sand ratio on flexural strength.

The flexural strength of the PBG grouting material exhibits a pronounced positive correlation with the binder-to-sand ratio (Figure 19). Experimental results indicate that as the binder-to-sand ratio increases from 0.60 to 0.75, the early-age (3 days) flexural strength significantly rises from 0.16 MPa to 0.87 MPa, marking an increase of 443%. In the long term (28 days), the flexural strength increases from 0.61 MPa to 2.41 MPa, representing a 295% increase. The comparison reveals that the enhancing effect of the binder-to-sand ratio is more prominent during the early stages of strength development. Elevated binder-to-sand ratios accelerate the hydration reaction of the PBG grouting material more effectively. As a result, a denser microstructure is formed at an early stage, which contributes to the improvement of initial mechanical properties.

**Figure 20:**
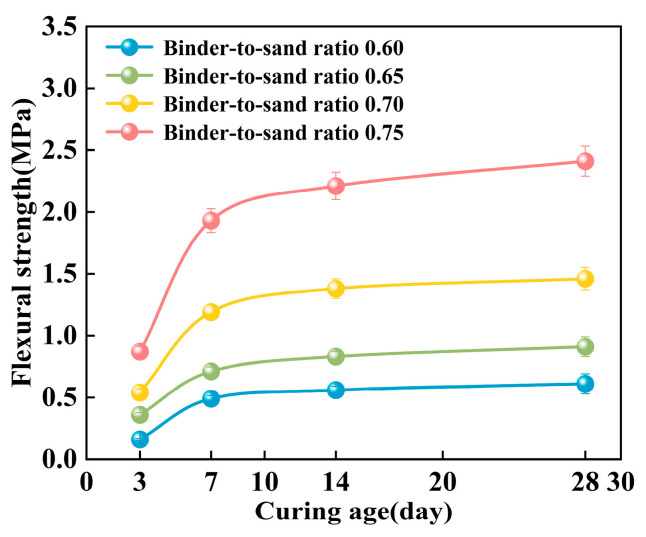
Effect of curing age on flexural strength.

As shown in Figure 20, the flexural strength of the PBG grouting material exhibits a pronounced time-dependent characteristic, displaying a progressive enhancement trend with increasing curing age. Experimental data indicate that the material exhibits rapid early-age strength development. The most significant increase in flexural strength occurred within the 3–7 days curing period. The 7 days flexural strength reaches approximately 2.0 to 3.1 times that of the 3 days strength. In contrast, strength development beyond 7 days tends to plateau, showing a more gradual growth trend. It is noteworthy that specimens with different binder-to-sand ratios exhibit distinct patterns of strength development. When the binder-to-sand ratio is 0.75, the long-term strength gain is significantly greater than that of specimens with a ratio of 0.60. This indicates that the binder content plays a crucial role in the long-term strength development of PBG grouting materials.

#### 3.2.3. Splitting Tensile Strength

**Figure 21:**
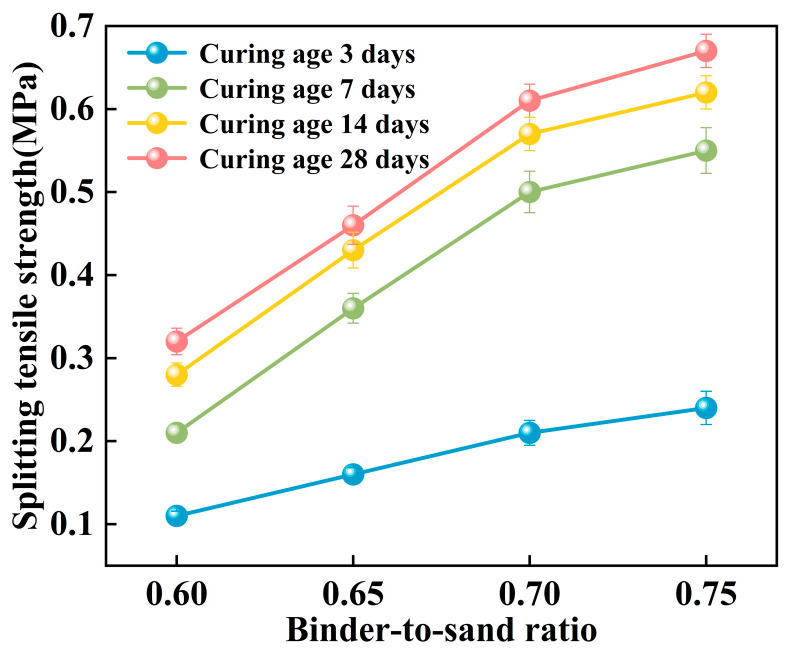
Effect of binder-to-sand ratio on splitting tensile strength.

The splitting tensile strength of the PBG grouting material exhibits a pronounced positive correlation with the binder-to-sand ratio (Figure 21). Experimental results indicate that when the binder-to-sand ratio increases from 0.60 to 0.75, the splitting tensile strength of the specimens rises significantly from 0.21 MPa to 0.55 MPa. An increase of 262%. This phenomenon can be attributed to the increased production of hydration gel resulting from the higher binder-to-sand ratio, which enhances the internal bonding strength of the material and subsequently improves its splitting tensile performance. The data confirm that appropriately increasing the binder-to-sand ratio has a positive effect on enhancing the mechanical properties of PBG grouting material.

**Figure 22:**
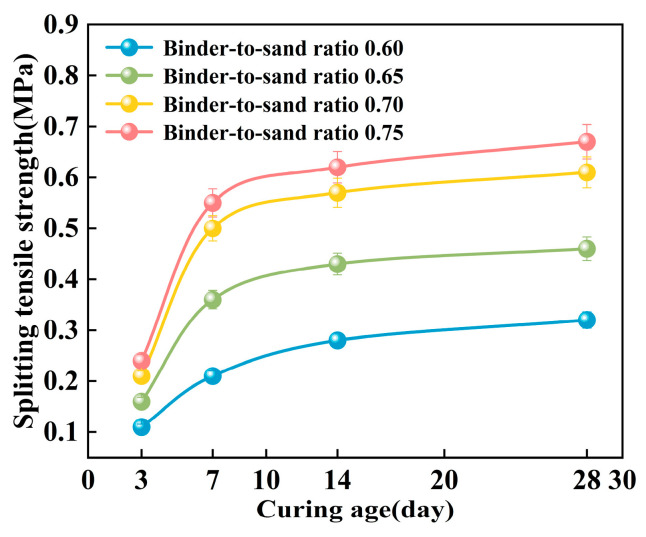
Effect of curing age on splitting tensile strength.

As shown in Figure 22, the splitting tensile strength of PBG grouting material exhibits a pronounced nonlinear growth trend with increasing curing age. Experimental results demonstrate that the strength development of the PBG grouting material follows a three-stage progression. A rapid increase in strength characterizes the early stage (0–7 days). This is followed by an intermediate stage (7–14 days), during which the growth rate slows significantly. In the later stage (after 14 days), the strength tends to stabilize, indicating the completion of major hydration and geopolymerization reactions. At varying binder-to-sand ratios, the 14 days splitting tensile strength reached 87.5%, 93.5%, 93.4%, and 92.5% of the corresponding 28 days values, respectively. This observation indicates that the geopolymerization reaction of the PBG grouting material exhibits high reactivity at the early stage, thereby significantly enhancing its early-age mechanical performance.

### 3.3. Durability of PBG Grouting Material

#### 3.3.1. Impermeability

**Figure 23:**
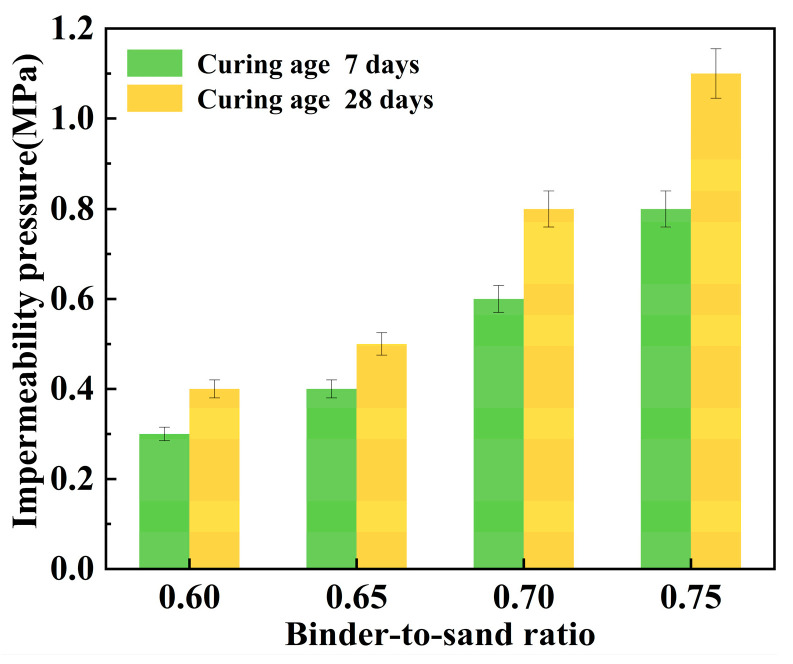
Effect of binder-to-sand ratio on impermeability pressure.

Figure 23 illustrates the relationship between the impermeability pressure of the PBG grouting material and the binder-to-sand ratio. It can be observed that the impermeability pressure increases with the increase in the binder-to-sand ratio. When the binder-to-sand ratio increased from 0.60 to 0.75, the 7 days impermeability pressure rose from 0.3 MPa to 0.8 MPa, representing an increase of 166.7%. Meanwhile, the impermeability pressure increased from 0.4 MPa to 1.1 MPa over 28 days, marking a 175% improvement. This improvement is primarily attributed to the increased dosage of PBG in the reaction system, resulting from the rise in the binder-to-sand ratio. The hydration products C-A-S-H, C-S-H, and AFt coexist and interweave to form a denser three-dimensional spatial structure. Moreover, the increased contact area between the slurry and sand particles significantly reduces internal porosity, thereby effectively impeding external water infiltration and enhancing the impermeability of the material. On the other hand, the presence of unreacted phosphogypsum and slag powder in the slurry may lead to synergistic interactions with sand particles. These interactions contribute to the formation of a dense skeletal gradation, effectively reducing the interparticle spacing. The connectivity of capillary pores is diminished, thereby enhancing the material’s compactness and stability. This further enhances the impermeability performance of the PBG grouting material. In addition, the impermeability pressure of the PBG grouting material exhibits an increasing trend as the curing period extends. This is primarily because prolonged curing promotes the continued progress of hydration reactions, thereby enhancing the impermeability of the specimens.

#### 3.3.2. Freeze–Thaw Resistance

**Figure 24:**
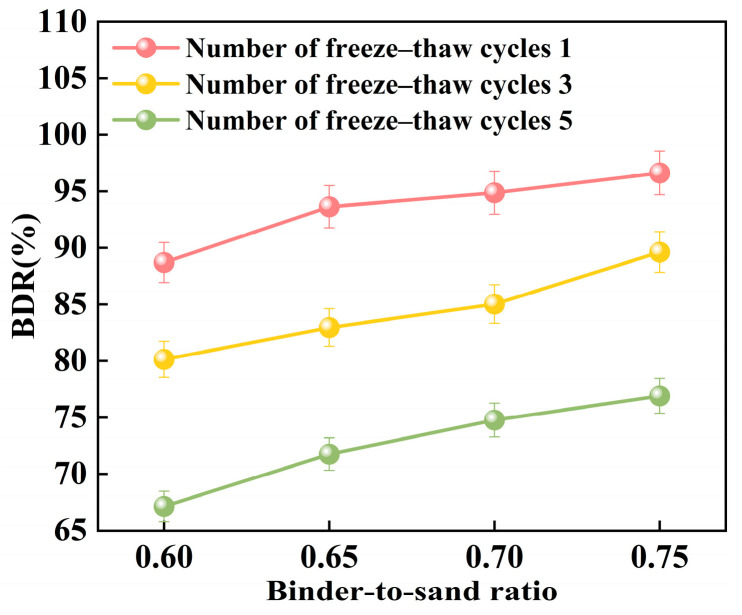
Effect of binder-to-sand ratio on BDR.

Figure 24 illustrates the relationship between the freeze–thaw resistance of PBG grouting material and the binder-to-sand ratio. As the binder-to-sand ratio increases, the BDR value of the PBG grouting material exhibits a gradual upward trend. Increasing the binder-to-sand ratio from 0.60 to 0.75 resulted in a notable improvement in the BDR of the PBG grouting material. Under 1, 3, and 5 freeze–thaw cycles, the BDR values increased from 88.70%, 80.14%, and 67.12% to 96.63%, 89.62%, and 76.90%, respectively. These results indicate that a higher binder content significantly enhances the material’s resistance to freeze–thaw deterioration. This improvement is primarily attributed to the increased binder-to-sand ratio, resulting in a higher content of phosphogypsum and slag powder particles in the slurry. Since both possess smaller particle sizes compared to sand, the overall porosity of the PBG grouting material is reduced, thereby enhancing its compactness. In addition, the geopolymerization process yields a greater quantity of hydration gel products. These gels contribute to stronger interfacial bonding among particles, which plays a crucial role in enhancing the material’s structural integrity.

**Figure 25:**
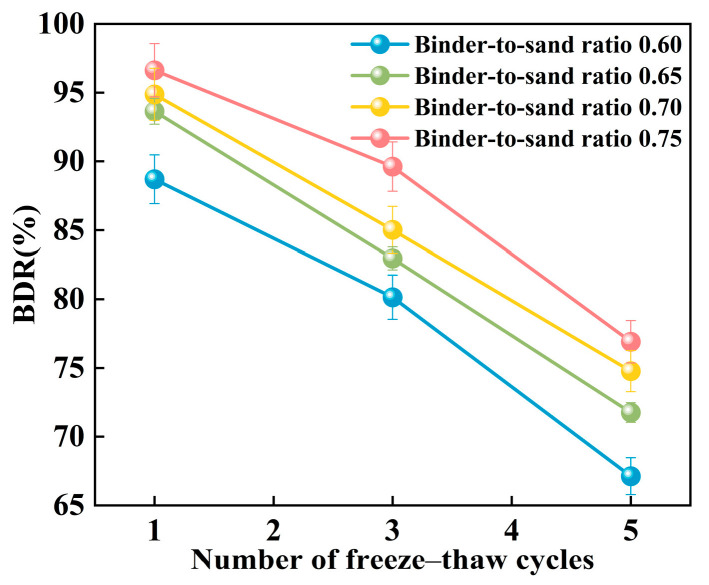
Effect of freeze–thaw cycles on BDR.

Figure 25 presents the relationship between the BDR of PBG grouting material and the number of freeze–thaw cycles. The BDR decreases significantly with the increasing number of freeze–thaw cycles. This phenomenon can be attributed to the freezing of absorbed water within the specimens when the temperature drops below 0 °C, resulting in volumetric expansion. When the resulting expansion stress exceeds the internal bonding strength of the grouting material, microcracks begin to form. Under repeated freeze–thaw cycles, the internal microcracks within the PBG grouting material are continuously subjected to infiltration, solidification, and thawing of free water. This process gradually widens the initially formed microcracks, thereby compromising the material’s structural integrity and leading to a reduction in strength.

### 3.4. Leaching of Toxic Substances from PBG Grouting Material

**Figure 26:**
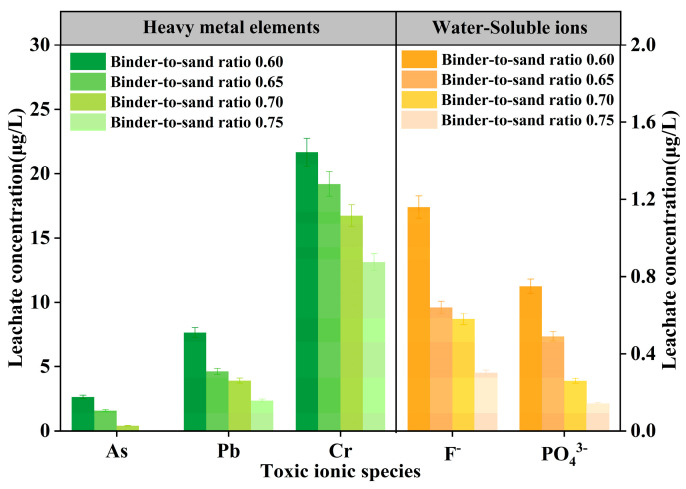
Leaching concentration of toxic ions in PBG grouting material.

The test results of As, Pb, Cr, F^−^, and PO_4_^3−^ in PBG grouting materials with different binder-to-sand ratios (0.60, 0.65, 0.70, and 0.75) are presented in Figure 26. The experimental results show that at a binder-to-sand ratio of 0.60, the leaching concentrations of heavy metals (As, Pb, and Cr) are 2.64 μg/L, 7.65 μg/L, and 21.67 μg/L, respectively. All measured values fall within the limits specified by the Class III groundwater quality standard. Additionally, the leaching concentration of water-soluble F^−^ is 1.16 mg/L, meeting the requirements of the Class IV groundwater quality standard. It is noteworthy that when the binder-to-sand ratio is increased to 0.75, the leaching concentrations of all toxic ions exhibit a declining trend. Specifically, arsenic (As) was not detected. The concentrations of lead (Pb) and fluoride (F^−^) decreased to 2.36 μg/L and 0.30 mg/L. Meeting the Class II groundwater quality standard. The leaching concentration of chromium (Cr) was reduced to 13.13 μg/L, still in compliance with the Class III groundwater quality standard, and the concentration of phosphate (PO_4_^3−^) dropped to 0.14 mg/L. This phenomenon can be attributed to the increased binder-to-sand ratio, which promotes the formation of hydration gel products and enhances the densification of the internal three-dimensional network structure. The synergistic effects of physical adsorption and encapsulation effectively inhibit the leaching behavior of ions.

### 3.5. Strength Development Mechanism and Immobilization Mechanism of Toxic Substances in PBG Grouting Material

**Figure 27:**
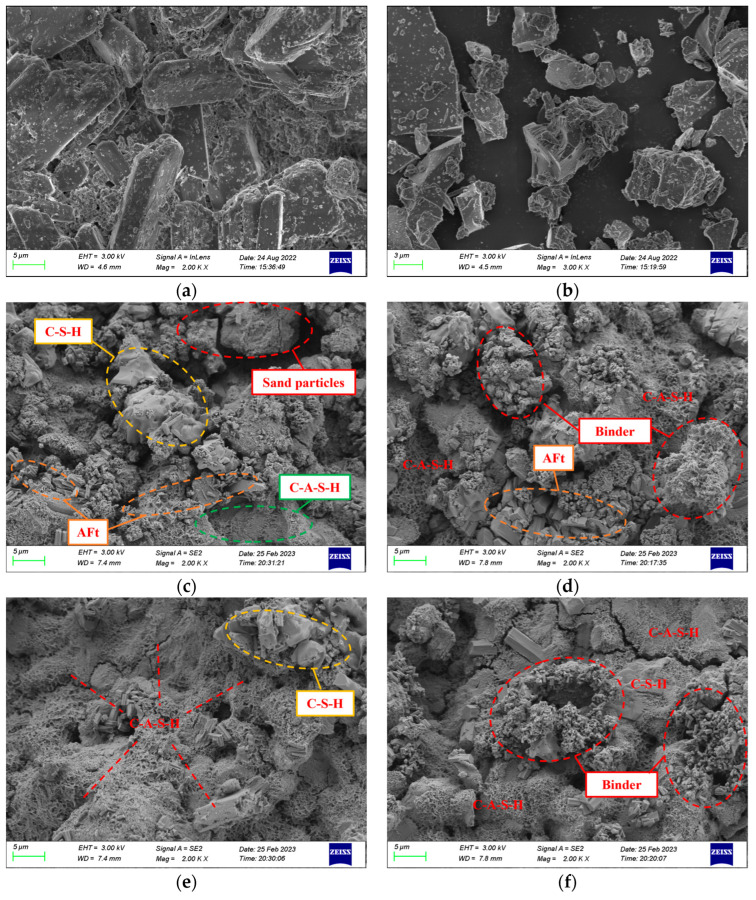
Microstructural morphology of materials (×2000, SEM): (**a**) Phosphogypsum (**b**) Slag (**c**) PBG grouting material with a binder-to-sand ratio of 0.60 (**d**) PBG grouting material with a binder-to-sand ratio of 0.65 (**e**) PBG grouting material with a binder-to-sand ratio of 0.70 (**f**) PBG grouting material with a binder-to-sand ratio of 0.75.

SEM was employed to investigate the microstructure of phosphogypsum and slag, as well as PBG grouting specimens that had been cured for 28 days under different binder-to-sand ratios. The results are presented in Figure 27.

As shown in Figure 27a, the main mineral phase of phosphogypsum is CaSO_4_·2H_2_O crystals. The crystals mainly appear as irregular plate-like structures. They are shaped as rhombohedra, rectangles, and parallelograms. The crystals show a high degree of crystallinity. Their edges are sharp and clearly visible. The particle size distribution is uneven. Crystals overlap and cross each other, creating numerous surface pores. Small crystal fragments and impurities are also attached. According to Figure 27b, slag shows an irregular clastic morphology. The particles resemble crushed-stone grains with sharp edges. The distribution is relatively uniform. The surface is dense and shows no noticeable pores. However, fine particles and other impurities adhere to the surface. Microstructural analysis of Figure 27c indicates that at a binder-to-sand ratio of 0.60, the amorphous flocculent gel products within the PBG grouting material are sparsely distributed. Some sand particles remain insufficiently encapsulated, resulting in a relatively high porosity and a loosely structured internal matrix. In contrast, Figure 27d shows that when the binder-to-sand ratio is increased to 0.65, the amount of gel products increases significantly. Sand particles are fully encapsulated, and some fine gel particles aggregate to form larger gel masses. Additionally, the formation of columnar AFt hydration products is observed, effectively filling the interparticle voids and contributing to a denser internal structure of the material. Further observations of Figure 27e,f reveal that when the binder-to-sand ratio is increased to 0.70 and 0.75, the surface of the PBG grouting material is extensively covered with C-A-S-H and C-S-H gels, along with AFt crystals. The hydration products interweave to form a continuous three-dimensional network structure. This structural framework enhances interfacial bonding among particles and significantly reduces internal porosity. Therefore, the macroscopic mechanical performance of the material is markedly improved. In addition, the immobilization mechanism of toxic ions in PBG grouting material involves multiple synergistic processes:

1. Physical adsorption: toxic ions are immobilized through surface adsorption and encapsulation by the gel matrix.
2. Chemical substitution: heavy metal ions such as As^5+^, Pb^2+^, and Cr^3+^ are incorporated into the gel lattice by substituting for Al^3+^.
3. Precipitation reaction: water-soluble ions such as F^−^ and PO_4_^3−^ react with Ca^2+^ in the system to form insoluble compounds, thereby achieving the stabilization and immobilization of toxic ions.

### 3.6. Carbon Footprint Analysis

To comprehensively assess the environmental benefits of PBG grouting material, the Life Cycle Assessment (LCA) approach was employed to evaluate its environmental impact systematically. In this analysis, PBG grouting material with a binder-to-sand ratio of 0.75 was selected as a representative case. Ordinary Portland Cement (OPC) grouting material was modeled using a water-to-cement ratio of 0.5 and a cement dosage of 400 kg/m^3^. A transportation distance of 100 km was assumed for both systems.

The analysis results (Table 4) indicate that CO_2_ emissions during the production phase constitute the primary environmental burden. The Global Warming Potential (GWP) of PBG grouting material is 120 kg CO_2_ eq/m^3^, representing a 67% reduction compared to conventional OPC-based grouting material. The primary contribution to carbon emission reduction originates from the low-carbon characteristics of phosphogypsum and slag. However, the production of alkali activators represents the primary source of the carbon footprint. It accounted for 54% of the total carbon emissions of the PBG grouting material. This highlights the broad significance of LCA in evaluating circular economy strategies for road materials.

In fact, extending the discussion beyond this system, the LCA framework is also essential for evaluating other forms of low-carbon recycling. For example, the utilization of blast furnace slag to produce zeolite-based geopolymer building materials [37] and sulfuric acid co-production cement (PSC) from phosphogypsum [38] has both been demonstrated to reduce CO_2_ emissions significantly. Such approaches can further enhance the high-value utilization of industrial solid wastes such as slag and phosphogypsum, while reducing the carbon footprint of the construction and industrial sectors.

In summary, PBG grouting material achieves a reduction in carbon emissions while maintaining excellent mechanical performance, demonstrating clear advantages in environmental sustainability. This material has significant application potential in the fields of solid waste valorization and the development of low-carbon construction materials.

### 3.7. Application Recommendation

This study systematically evaluated the workability (flowability, setting time, and bleeding rate), mechanical strength (compressive strength, flexural strength, and splitting tensile strength), durability (impermeability and freeze–thaw resistance), and leaching behavior of hazardous substances in PBG grouting material. Furthermore, a quantitative carbon footprint analysis was conducted based on the LCA methodology. The results indicate that the PBG grouting material demonstrates excellent workability at a water-to-solid ratio of 0.28. Specifically, the flowability ranges between 160 mm and 240 mm, the initial setting time exceeds 30 min, the final setting time remains under 400 min, and the bleeding rate stays below 0.40%. All these performance indicators are in full compliance with relevant engineering specifications, confirming the suitability of this mix proportion for practical applications. Further investigation into the influence of PBG content on the mechanical properties of the grouting material revealed a substantial strength enhancement when the binder-to-sand ratio was maintained between 0.70 and 0.75. Under these conditions, the 28 days compressive strength consistently exceeded 9.0 MPa, significantly surpassing the minimum design requirement of 4.2 MPa specified for road grouting materials. In addition, leaching tests of hazardous substances indicate that the concentrations of heavy metal components such as As, Pb, Cr, and F^−^ in the PBG grouting material are all below the Class III groundwater quality standard limits. Notably, the maximum leachate concentration of PO_4_^3−^ is 0.26 mg/L, suggesting that the material poses a controllable risk to the groundwater environment.

In summary, the PBG grouting material was comprehensively evaluated in terms of workability, mechanical strength, durability, and environmental safety. The results indicate that it exhibits optimal overall performance when the water-to-solid ratio is set at 0.28. The binder-to-sand ratio should range between 0.70 and 0.75 to achieve the best performance. This mix design satisfies the technical requirements for road grouting applications. It also demonstrates strong potential for trenchless repair in a wide range of road engineering projects.

## 4. Conclusions

In this study, PBG was employed as the primary binder material and combined with fine sand to prepare a PBG grouting material. A series of laboratory experiments were conducted to systematically investigate the influence of multi-factor coupling effects on the workability, mechanical strength, durability, and leaching behavior of toxic substances. Additionally, microstructural characterization was conducted to elucidate the mechanism of strength development and the immobilization behavior of hazardous ions. An environmental impact assessment was also carried out to evaluate the effectiveness of heavy metal immobilization and the overall environmental performance of the material. Furthermore, a carbon footprint analysis was performed. The experimental results indicate that

1. The water-to-solid ratio and binder-to-sand ratio strongly influence the workability of PBG grouting material. Increasing the water-to-solid ratio improves the slurry’s flowability, prolongs the setting time, and increases the bleeding rate. In contrast, an increase in the binder-to-sand ratio produces the opposite trends. When the water-to-solid ratio is set to 0.28 and the binder-to-sand ratio is maintained within the range of 0.70 to 0.75, the slurry exhibits optimal overall workability, meeting the technical standards required for grouting applications in trenchless road rehabilitation.
2. The mechanical properties of PBG grouting material (compressive strength, flexural strength, and splitting tensile strength) show a significant positive correlation with both the binder-to-sand ratio and curing age. The minimum compressive strength at 28 days reaches 5.84 MPa, meeting the technical requirements for trenchless road rehabilitation projects.
3. The impermeability of PBG grouting material increases with an increasing binder-to-sand ratio. At a ratio of 0.75, the 7 days impermeability pressure reaches 0.8 MPa, indicating that the material exhibits excellent impermeability at higher binder-to-sand ratios. Additionally, the frost resistance of the PBG grouting material improves with an increase in the binder-to-sand ratio. After five freeze–thaw cycles, the material’s BDR remains above 65% across all specimens, demonstrating good freeze–thaw durability.
4. At a binder-to-sand ratio of 0.75, arsenic (As) was not detected in the leachate of the PBG grouting material, and the concentrations of lead (Pb) and fluoride (F^−^) complied with the Class II groundwater quality limits specified in the Standard [29]. Chromium (Cr) met the Class III standard, and the leachate concentration of phosphate (PO_4_^3−^) was reduced to 0.14 mg/L. These results indicate that the material poses no pollution risk to the groundwater environment.
5. The strength development of the PBG grouting material is principally attributed to the establishment of a dense three-dimensional network structure. This structure is the consequence of a synergistic interaction between amorphous gel phases (C-A-S-H and C-S-H) and crystalline phases (Aft). Additionally, the gel particles improve matrix compactness via pore-filling effects. The immobilization mechanism of toxic ions in PBG grouting material involves physical adsorption and encapsulation, chemical substitution, and precipitation fixation. The gel phases restrict ion migration through surface adsorption and pore blockage. Ca^2+^ participates in ion exchange with heavy metal ions. Harmful ions react with matrix components to form insoluble compounds, thereby significantly reducing their leaching potential.
6. Based on the LCA evaluation, PBG grouting material demonstrates significant environmental benefits. Compared to OPC-based grouting material, it exhibits a notable 67% reduction in GWP. This advantage is primarily attributed to the low-carbon characteristics of phosphogypsum and slag, offering an effective solution for promoting environmental sustainability.
7. PBG grouting material performs best overall when used with a water-to-solid ratio of 0.28 and a binder-to-sand ratio between 0.70 and 0.75. It exhibits both excellent mechanical and durability properties and is environmentally friendy. These characteristics highlight its strong potential for use in engineering applications for trenchless road rehabilitation.

## Figures and Tables

**Table 1:** Composition of PBG.

Phosphogypsum: Slag	Alkali Activator Dosage (%)	Alkali Activator Modulus	Water-to-Solid Ratio
1:1	16	1.2	0.52

**Table 2:** Key technical properties of PBG.

Compressive Strength at Different Ages (MPa)			Leaching Concentration of Toxic Ions				
3 days	7 days	28 days	As (μg/L)	Pb (μg/L)	Cr (μg/L)	F^−^ (mg/L)	PO_4_^3−^ (mg/L)
21.38	23.53	24.16	0.58	1.67	4.84	0.78	0.035

**Table 3:** Design of experimental variables and levels.

Testing Content		Variables	Levels
Workability		binder-to-sand ratio	0.60, 0.65, 0.70, 0.75
		water-to-solid ratio	0.26, 0.28, 0.30, 0.32
Mechanical strength		binder-to-sand ratio	0.60, 0.65, 0.70, 0.75
		curing age	3 days, 7 days, 14 days, 28 days
Durability	Impermeability	binder-to-sand ratio	0.60, 0.65, 0.70, 0.75
		curing age	7 days, 28 days
	Freeze–thaw resistance	binder-to-sand ratio	0.60, 0.65, 0.70, 0.75
		number of freeze–thaw cycles	1, 3, 5
Leaching of toxic substances		binder-to-sand ratio	0.60, 0.65, 0.70, 0.75

**Table 4:** GWP comparison.

Emission Stages	PBG Grouting Material (kg CO_2_ eq/m^3^)	OPC-Based Grouting Material (kg CO_2_ eq/m^3^)
Binder/Cement production	21	320
Alkali activator production	65	-
Aggregate transportation	18	18
Mixing and placement stage	16	12
Total	120	350

## Data Availability

The datasets presented in this article are not readily available because the data are part of an ongoing study.
